# Delayed Right Diaphragmatic Hernia With Chilaiditi Syndrome: A Case Report

**DOI:** 10.7759/cureus.41420

**Published:** 2023-07-05

**Authors:** Bayley Richardson, Leigh Hickham, Shane Harper, Basem Soliman

**Affiliations:** 1 Surgery, Texas Tech University Health Sciences Center School of Medicine, Amarillo, USA; 2 Dermatology, Louisiana State University Health Sciences Center, New Orleans, USA; 3 Surgery, Texas Tech University Health Sciences Center, Amarillo, USA

**Keywords:** abdominal pain, hernia, trauma, incarcerated liver, delayed presentation of diaphragmatic hernia, chilaiditi sign, chilaiditi syndrome, right-sided diaphragmatic hernia

## Abstract

Diaphragmatic hernias can be congenital or acquired and manifest as a defect thus allowing abdominal contents to protrude into the thorax through the defect. Common presentations and symptoms can include shortness of breath, nausea, vomiting, and abdominal pain. Rarely colon or small bowel is interposed between the liver and the diaphragm, Chilaiditi sign. When the Chilaiditi sign is accompanied by symptoms it is termed Chilaiditi syndrome. We present a case of a 41-year-old male who was involved in a motor vehicle accident 12 years prior and presented with a right diaphragmatic hernia and Chilaiditi syndrome. The patient presented with a 21-hour history of abdominal pain, nausea, and vomiting. A computed tomography scan of the chest and abdomen revealed the presence of Chilaiditi sign, wherein the large bowel was positioned above the liver, having herniated through a diaphragmatic defect. The patient subsequently underwent an exploratory laparotomy which confirmed an 8 x 4 cm right diaphragmatic defect. Primary repair was completed with intraperitoneal mesh. Diaphragmatic hernias pose diagnostic challenges due to their variable symptomatology and possible delayed onset. Consequently, the importance of including diaphragmatic hernia as part of the differential diagnoses for patients experiencing abdominal pain and/or difficulty breathing is highlighted by this case, especially for individuals with a distant record of trauma.

## Introduction

Diaphragmatic hernias can be of either congenital or acquired etiology. These hernias manifest as a defect in the diaphragmatic structure that allows abdominal contents such as loops of the bowel, liver, or spleen to protrude through its orifice. While the diaphragmatic injury is relatively infrequent, up to 5% of diaphragmatic hernias are attributable to thoracoabdominal blunt and penetrating traumas [1-3]. The anatomical position and size of the liver make right-sided hernias less likely, hence the majority of hernias present on the left. Clinically, patients with diaphragmatic hernias may present with diverse symptoms, including but not limited to shortness of breath, chest pain, tachypnea, and abdominal pain [1].

In addition, there exists an even rarer phenomenon known as the Chilaiditi sign, an anatomical anomaly where the intestine lies between the liver and the right hemidiaphragm. When this anomaly is accompanied by symptoms such as abdominal pain, nausea, vomiting, and respiratory distress, it is termed Chilaiditi syndrome. This sign was initially described by Demetrius Chilaiditi in 1910 after identifying the bowel between the liver and diaphragm in the chest and abdominal radiographs. Within the general population, the incidence of the Chilaiditi sign ranges from 0.025% to 0.28% and is observed more commonly in males [4]. The low frequency of this abnormality is due to the typical fixation of the colon by suspensory ligaments. We present a case of a 41-year-old male presenting 12 years status post motor vehicle accident with a right diaphragmatic hernia and Chilaiditi syndrome repaired via urgent exploratory laparotomy.

## Case presentation

**Figure 1:**
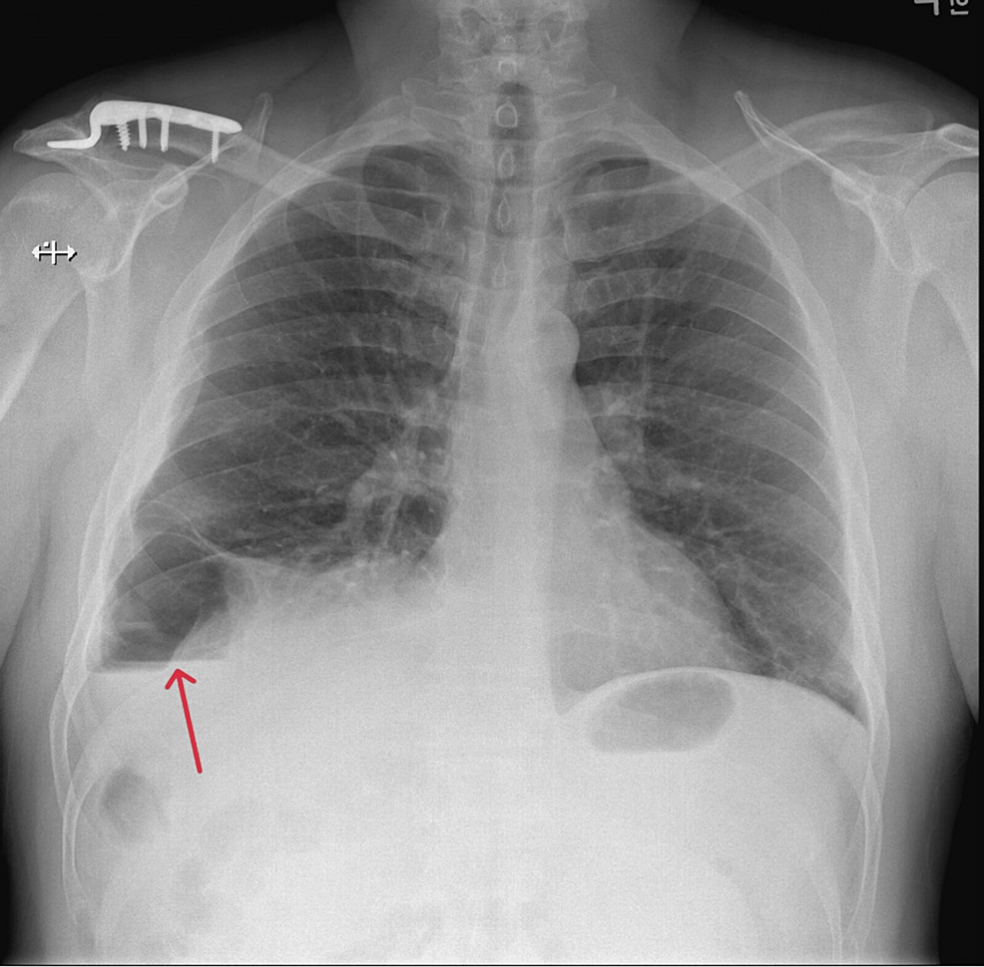
Chest x-ray showing ill-defined right lung base. Red Arrow: air-fluid level

A 41-year-old man presented to the emergency department with a 21-hour history of abdominal pain with radiation to his back and vomiting. He reported the pain was initially diffuse but then intensified and localized to the right up quadrant. During this time, he was anorexic to food or drink due to nausea and vomiting, and denied bowel movements or flatus. Medical history included a motor vehicle accident 12 years before and class I obesity (BMI 34.97 kg/m^2^). Initial vital signs: blood pressure 153/107 mm Hg; pulse of 61 beats/min; respiratory rate of 19 breaths/min; SpO_2_ at 97% on room air; temperature at 36.5℃. He did not appear in acute distress. Lung exam revealed non-labored respirations that were clear to auscultation and equal bilaterally and cardiac auscultation revealed no murmurs, rubs, or gallops. Inspection of the abdomen showed no distention, masses, hernias, or cicatrix. Palpitation of the abdomen demonstrated diffuse tenderness. Initial laboratory tests revealed elevated alanine transaminase (ALT) 74 units/L (16-63 units/L) and aspartate aminotransferase (AST) 52 units/L (15-37 units/L). Other labs within complete blood count, complete metabolic panel, and urinalysis were within normal limits. Chest x-ray showed ill-defined right lung base opacities and suspected air-fluid level within the right hemithorax (Figure 1).

**Figure 2:**
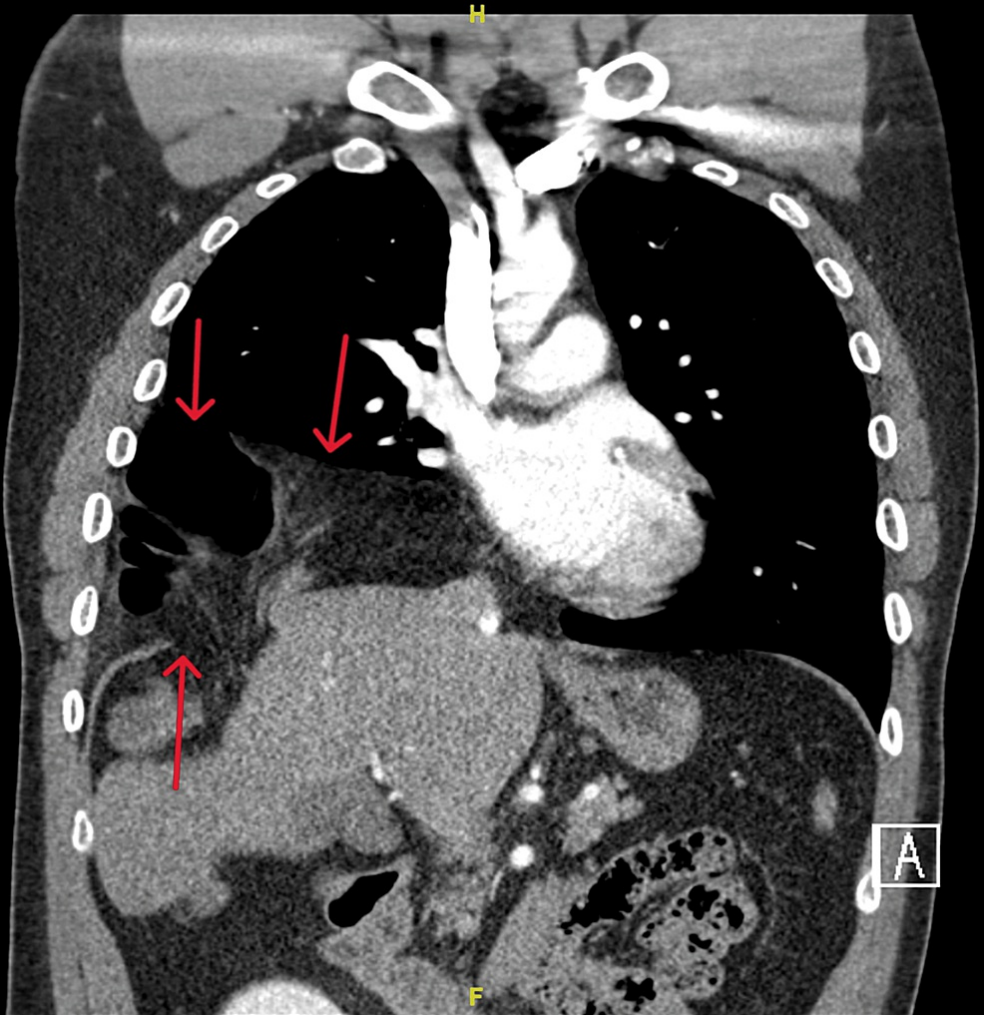
Coronal chest/abdomen CT showing right diaphragmatic hernia with incarcerated large bowel and liver and Chilaiditi sign. Red Arrows: Large Bowel

**Figure 3:**
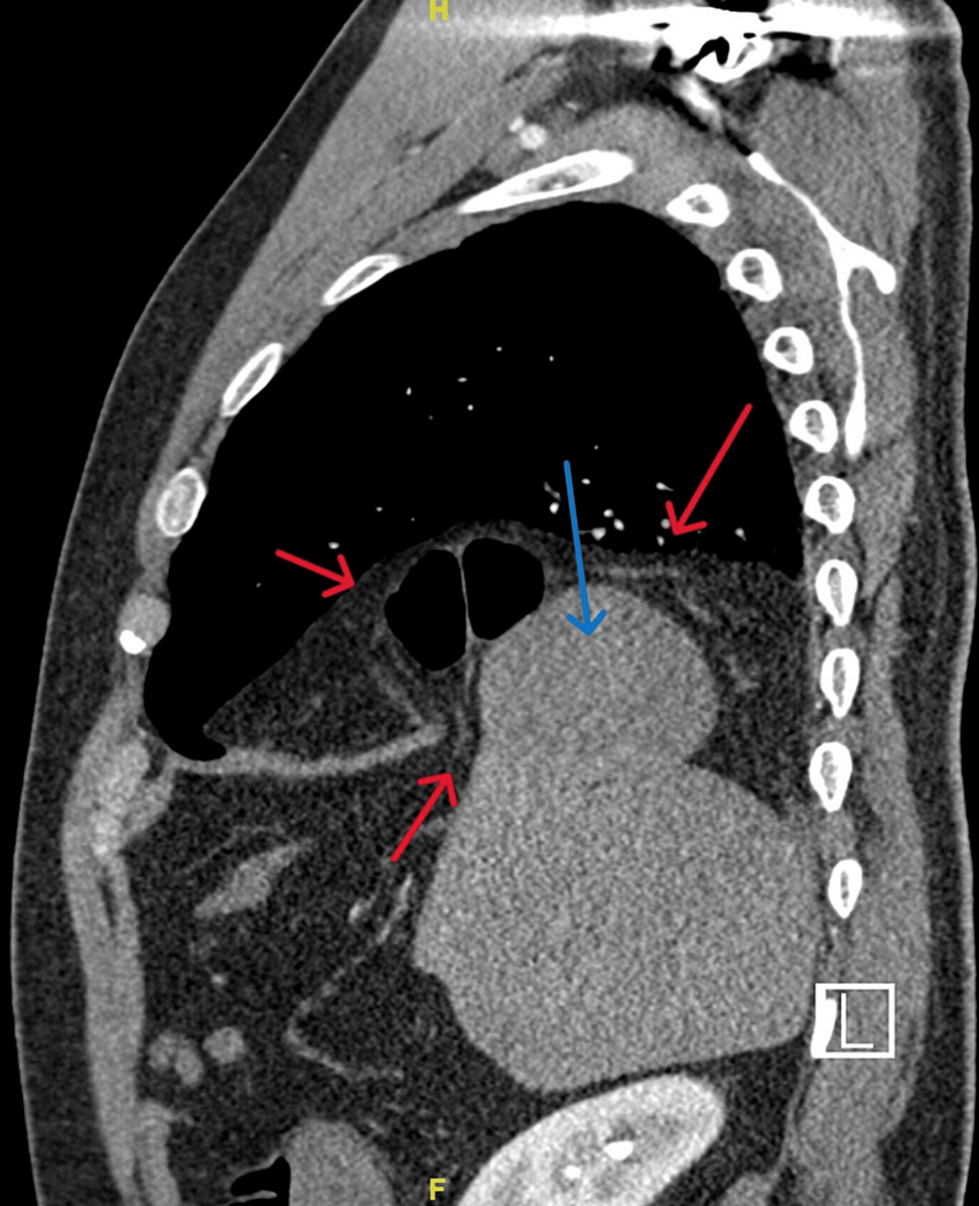
Sagittal chest/abdomen CT showing right diaphragmatic hernia with Chilaiditi sign. Red Arrows: Large Bowel, Blue Arrows: Liver

**Figure 4:**
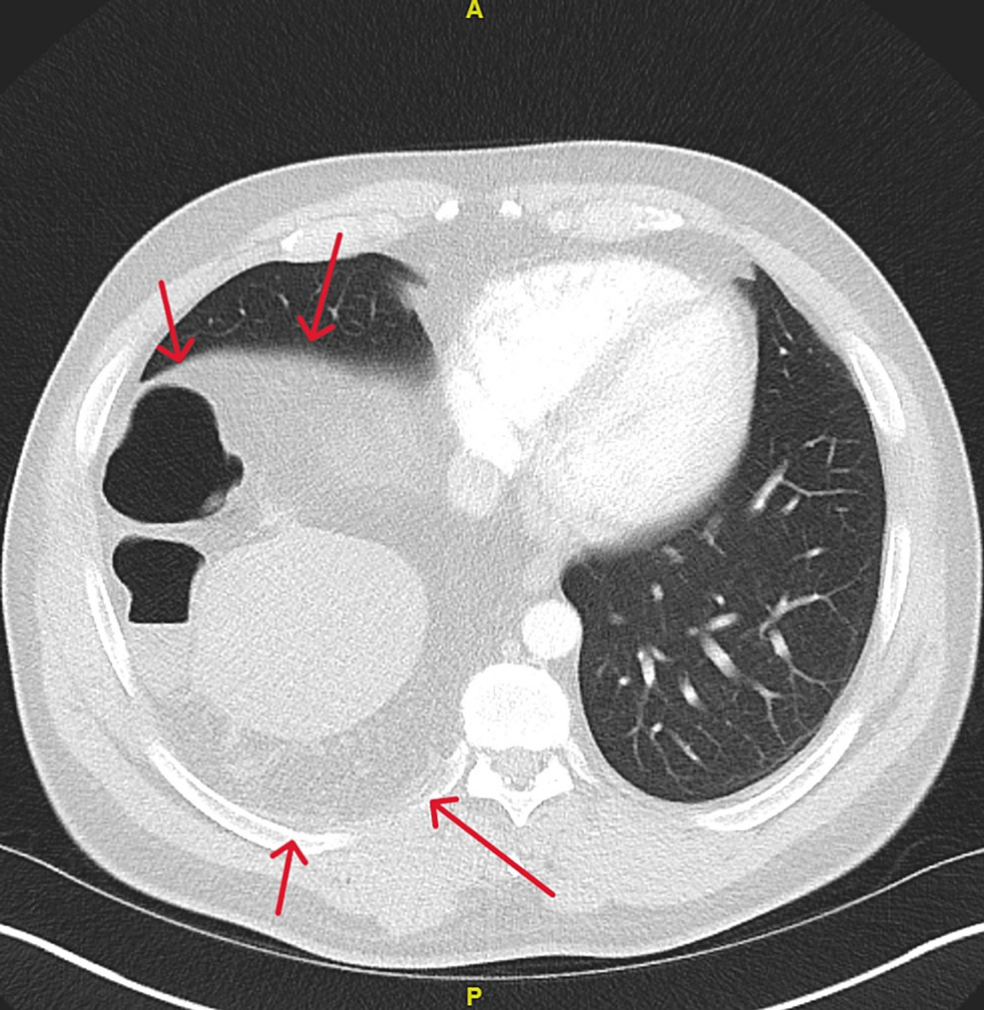
Chest CT showing liver and large bowel within the right lung field. Red Arrows: Large bowel

CT chest/abdomen demonstrated a right diaphragmatic defect measuring up to 4 cm and contained large bowel at the hepatic flexure. There was decompression of the large bowel distal to the diaphragmatic hernia, which represented possible obstruction, and herniation of liver superiorly (Figures 2-4). Figures 2, 3 illustrate the presence of Chilaiditi sign, characterized by the abnormal positioning of the large bowel above the liver. Based on the patient's manifestation of abdominal pain and vomiting, along with the radiographic identification of Chilaiditi sign, a diagnosis of Chilaiditi syndrome was established.

The patient was taken to the operating room where an exploratory laparotomy was completed. Intraoperatively, an 8 x 4 cm defect in the right posterior diaphragm was identified with large bowel, omentum, and liver herniating into the right hemithorax. Electrocautery was utilized to extend the defect laterally to allow for reduction of the incarcerated contents. Contents were reduced back into the abdominal cavity with no evidence of vascular compromise or necrosis. Primary repair of the defect was achieved using an #0 STRATAFIX™ Symmetric PDS™ Plus (Ethicon Inc, J & J Company; Bridgewater, NJ) and was reinforced with a 12 x 8 cm Gore® Synecore Biomaterial (W. L. Gore & Associates, Inc; Flagstaff, AZ) intraperitoneal mesh. A right-sided #32 French thoracostomy tube was placed at the end of the surgical procedure. The patient was extubated and recovered in a common fashion before being transported to the surgical intensive care unit in stable condition.

Patient had an uncomplicated postoperative course. Chest tube was removed on postoperative day two, and patient was discharged home on postoperative day four. At the two-week postoperative follow-up, patient denied any recurring symptoms.

## Discussion

Chilaiditi sign is an uncommon radiological finding in which the intestine lies between the right hemidiaphragm and the liver. The radiographic features include the presence of air below the diaphragm, which can be misinterpreted as pneumoperitoneum. The differentiating factor is that air levels do not change based on the patient's positioning in Chilaiditi sign [5]. Typically, this is an incidental finding that does not require further treatment, but when accompanied by symptoms, it is termed Chilaiditi syndrome. The presentation of Chilaiditi syndrome varies from constipation to marked chest pain [5-7]. Conservative management with a nasogastric tube, bowel rest, and fluid resuscitation is appropriate for the majority of patients, while up to one-fourth of patients may require surgical intervention [7-9]. Therefore, Chilaiditi Syndrome is an important differential diagnosis when radiological studies reveal pneumoperitoneum.

Acquired diaphragmatic hernias represent a rare pathologic condition that requires a timely diagnosis and management to avoid detrimental sequela. The majority of cases arise from high velocity (acceleration/deceleration) thoracoabdominal trauma, and their presentation can be challenging to decipher due to the variability of symptom onset. This form of hernia may present with diverse symptoms such as abdominal pain, nausea, vomiting, chest pain, and dyspnea. The left hemidiaphragm is more susceptible to herniation events due to the protective effect of the liver on the right side [1-3,10,11]. Right-sided hernias tend to be associated with higher morbidity and mortality rates [12]. The contents of the abdominal cavity, including the stomach, liver, large and small bowel, omentum, and spleen may herniate through the diaphragmatic defect. Failure to diagnose diaphragmatic hernias may lead to a range of complications, including respiratory insufficiency, vascular compromise leading to bowel strangulation and necrosis, and cardiac tamponade [10].

Chest x-rays are commonly the first radiological study ordered with a suspected diaphragmatic hernia, but only 25% to 49% of initial x-rays reveal diagnostic findings, such as abdominal contents within the thorax [13-14]. Signs on x-ray that suggest diaphragmatic hernia include loops of bowel within the thorax, elevated hemidiaphragm, nasogastric tube above the hemidiaphragm, but these findings may mimic diaphragmatic masses and lipomas [15]. Computed tomography (CT) is a superior imaging modality due to the ability to visualize the defect in multiple planes. Even with the increased sensitivity and specificity provided by CT imaging, right sided hernias are often missed on imaging [14,16]. Common CT manifestations include the “collar sign,” which is a constriction of the herniated structure at the level of the diaphragmatic defect. The “hump sign” may also be identified, which corresponds to the herniated liver at the level of the diaphragm [9]. 

In 1951, Carter et al. provided an account of the natural progression of traumatic diaphragmatic hernias, delineating three distinct stages [17]. The first stage is the acute phase, which is the time immediately following the injury. The patient then enters the latent phase where they may or may not have symptoms. The obstructive phase follows and includes herniated bowel becoming incarcerated or strangulated [17]. These phases represent a critical framework for understanding the evolution of diaphragmatic hernias and are essential in guiding the differential diagnosis in a patient presenting with a recent or past history of trauma.

## Conclusions

Right-sided diaphragmatic hernias are a rare but potentially life-threatening condition that can be of either congenital or acquired etiology. Acquired diaphragmatic hernias are commonly associated with high-velocity thoracoabdominal trauma and may present with diverse symptoms. Further, awareness of the Chilaiditi sign is important when interpreting radiographic studies revealing pneumoperitoneum. Conservative management can be sufficient, but surgical intervention may be required in certain cases. This case underscores the significance of including diaphragmatic hernia in the list of differential diagnoses for patients presenting with abdominal pain and/or dyspnea, particularly in those with a remote history of trauma.
